# National Surveillance of Home-Based HIV Testing Among Australian Gay and Bisexual Men, 2018–2020: Uptake After Commercial Availability of HIV Self-Tests

**DOI:** 10.1007/s10461-023-04124-x

**Published:** 2023-07-13

**Authors:** Ye Zhang, Martin Holt, Curtis Chan, Tanya L. Applegate, Benjamin R. Bavinton, Timothy R. Broady, Phillip Keen, Luh Putu Lila Wulandari, Limin Mao, Hamish McManus, Nicholas A. Medland, Garrett Prestage, Virginia Wiseman, Rebecca J. Guy

**Affiliations:** 1 Kirby Institute, UNSW Sydney, Wallace Wurth Building, High Street, Sydney, NSW 2031 Australia; 2 Centre for Social Research in Health, UNSW Sydney, Sydney, NSW Australia; 3 Department of Global Health and Development, London School of Hygiene and Tropical Medicine, London, UK

**Keywords:** HIV self-testing, HIV home testing, HIV/AIDS, Gay and bisexual men, Australia

## Abstract

HIV self-testing allows people to collect samples and test themselves at home, addressing known barriers to facility-based testing. We aimed to measure the uptake of home HIV testing among Australian gay and bisexual men (GBM). Using national cross-sectional data from the Australian Gay Community Periodic Surveys, we assessed trends in home HIV testing among non-HIV positive GBM between 2018 and 2020. Overall, the use of home HIV testing was low, but slightly increased during 2018–2020 (from 0.3 to 0.8%, RR = 1.54, 95%CI = 1.23–1.92, p-trend < 0.001). Testing at home was more likely among non-HIV-positive GBM who were born overseas and recently arrived in Australia, at higher risk of HIV, and infrequent HIV testers. Given the greater use of home testing by men at higher risk of HIV, recent migrants and infrequent testers, all priority groups in Australia’s HIV epidemic, we recommend increasing access to HIV self-testing to enhance uptake in these and other groups of GBM.

**Supplementary Information:**

The online version contains supplementary material available at 10.1007/s10461-023-04124-x.

## Introduction

Australia has set an ambitious target of virtual elimination of HIV transmission [1], reflecting global efforts by the Joint United Nations Programme on HIV/AIDS to end the AIDS epidemic by 2030 [2]. Increasing the uptake and frequency of HIV testing is a priority strategy to eliminate HIV. In Australia, HIV testing among gay and bisexual men (GBM) has increased in recent years, in line with guidelines recommending quarterly HIV testing [3, 4]. Yet, the uptake of HIV testing remains suboptimal among a range of subgroups, including men living in areas with small proportion of gay men, and GBM who report condomless sex with casual partners but are not taking PrEP [3, 5]. Also, mathematical modelling suggests that migrant GBM living in New South Wales are nearly three times more likely to be undiagnosed for HIV than Australian-born GBM [5, 6], with undiagnosed infections contributing disproportionately to transmission [7]. Innovative HIV testing approaches, including HIV self-testing, are needed to optimise HIV testing among all Australian GBM.

HIV self-testing enables GBM to test in the privacy of their own home, making the process more convenient and accessible [8]. The World Health Organization has encouraged all countries to use HIV self-testing as a supplement to facility-based testing[8], based on data from randomised controlled trials, which showed that HIV self-testing increases the frequency of HIV testing among GBM, particularly among those who test infrequently [9–12]. Before the first HIV self-test received regulatory approval in Australia, several studies and demonstration projects were conducted to provide local evidence on its benefit and capacity to increase access to the technology. The first was a randomised controlled trial among GBM called FORTH, which provided free access to HIV self-tests among 362 participants in Australia from 2013 to 2015 [9]. The second was an observational study among GBM in Queensland, involving free access to mailed HIV self-test kits among 794 participants from 2016 to 2018, and the third was a home sampling program which involved mailed or in person pick up of dried blood spot test kits, specimen collection at home and postal return to the laboratory among high-risk populations in New South Wales in 2017 [13]. During this period, unlicensed HIV self-test kits could also be purchased online from overseas [14, 15]. One finger-prick HIV self-test kit was approved for use in Australia in the end of 2018 and was made commercially available in a restricted way [16]. The uptake of HIV testing at home by GBM since the first HIV self-test became commercially available has not been formally analysed at a national level.

Measuring the uptake of HIV testing and where testing is conducted is an essential HIV programmatic indicator. Yet tracking the number of HIV self-test kits used at home is challenging as private sales figures of HIV self-test kits are not publicly available. Also, purchasing a kit does not always equate to testing. As scale up of HIV self-testing gains traction globally, understanding the uptake of HIV self-testing in a real-world setting is needed to reflect the market conditions and guide future implementation. The Gay Community Periodic Surveys are Australia’s largest HIV behavioural surveillance system of GBM, conducted in seven states and territories. The surveys provide a unique opportunity to measure the proportion of GBM in Australia who report testing for HIV at home. For the first time since HIV self-tests became commercially available in Australia, we used national survey data to examine trends in the use of home HIV testing by Australian GBM men. In addition, this study provides insights into the subgroups of GBM that have accessed HIV home testing during a period of restricted availability.

## Methods

### Gay Community Periodic Surveys

The Gay Community Periodic Surveys are an essential part of Australia’s behavioural surveillance system for HIV. The surveys use time-location sampling of GBM from seven Australian states or jurisdictions [17], annually in larger jurisdictions and biennially in smaller jurisdictions (Supplementary Table 1).

Participants are recruited through healthcare settings, gay festival events, or online recruitment through Facebook, dating apps, and community organisation websites. Eligible participants include those who identify as male, at least 16 years old, currently living in Australia, reporting sex with men in the past 5 years, and/or self-identified as gay or bisexual [17]. Participation is completely voluntary without reimbursement and consent is implied through return of the survey. The Gay Community Periodic Surveys have ethical approval from the UNSW Sydney Human Research Ethics Committee, and the participating community organisations, ACON and Thorne Harbour Health.

### Study Population

For the analysis of trends in home testing during 2018–2020, the study population included all non-HIV-positive participants in the survey, including GBM who reported that their last HIV test result was HIV negative, or their HIV status was unknown, or they had never tested before. As they are not indicated for ongoing antibody testing, we excluded HIV-positive men from the trend analyses.

**Fig. 1:**
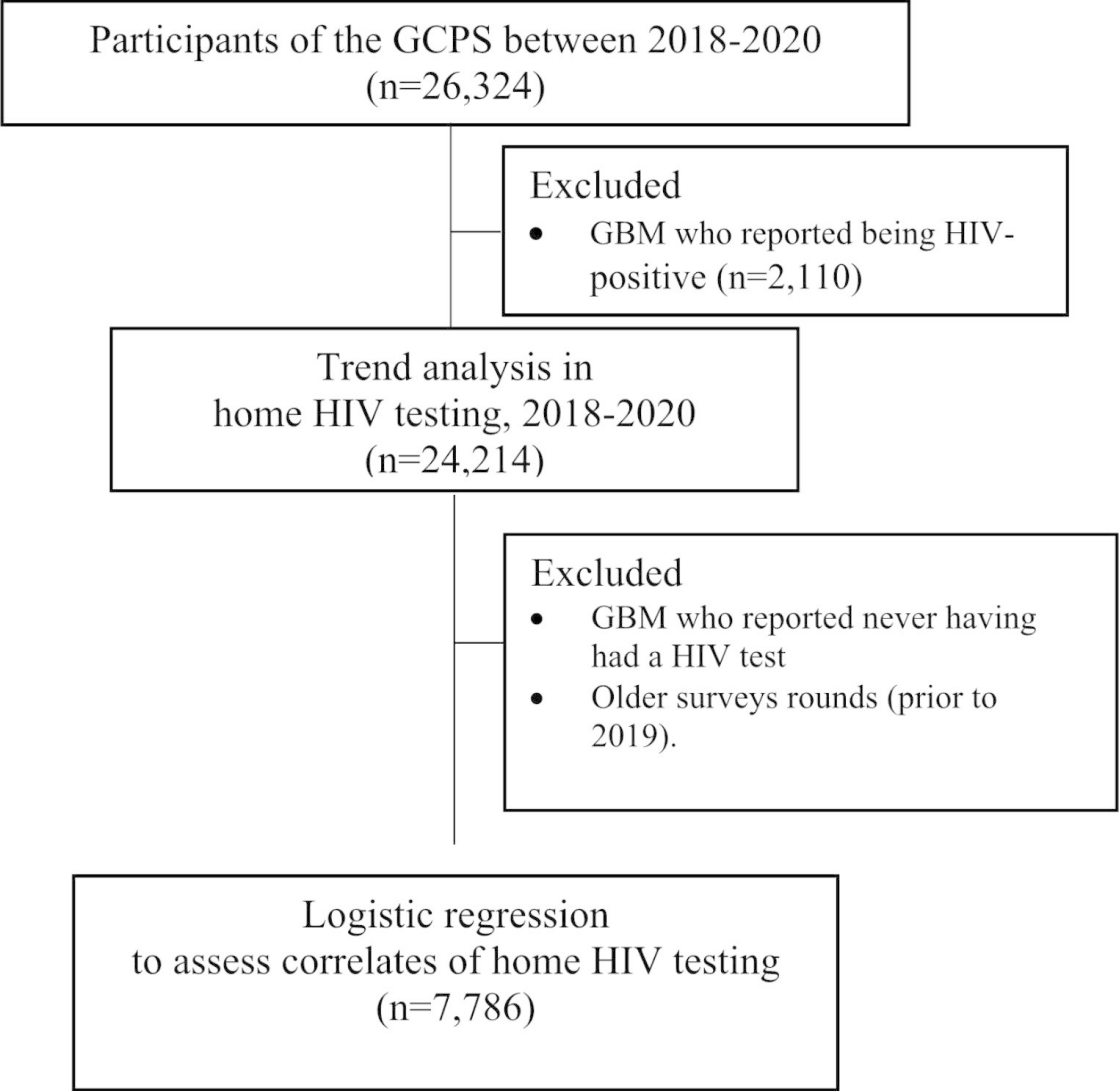
Flow chart of the study population for each analysis. GCPS = Gay Community Periodic Surveys; GBM = gay and bisexual men

For the analysis of correlates of home testing, the study population was further restricted to non-HIV positive men who had tested for HIV and completed the survey between 2018 and 2019, i.e. participants who had tested for HIV before and who had reported where their last HIV test occurred. Figure 1 shows the population included in the study.

### Study Outcome

The primary study outcome was ‘testing at home’ derived from a question asking participants where they last tested for HIV (‘Where did you have your last HIV test?’). Those who responded ‘tested at home’ were classified as home testers. Participants who reported testing at a general practice, sexual health clinic, hospital, or community-based service, were classified as testing at a health facility.

### Covariates

From the Gay Community Periodic Surveys, we selected a range of covariates informed by previous literature on GBM at higher risk of HIV, known gaps in HIV testing, and populations likely to benefit from HIV self-testing [6, 18, 19]. Participants were categorised by migrant status which was derived from the participant’s country of birth and recency of arrival in Australia. ‘Recent migrants’ were those who were born overseas and lived in Australia for *≤* 5 years. The length of time living in Australia was only available in the 2019 and 2020 surveys. Participants were also categorised according to the concentration of gay men living in the suburb they resided in based on their postcode (‘higher concentration of gay men’ ≥ 5% gay residents, versus ‘lower concentration of gay men’ <5% gay residents), as defined previously [20].

We also created the following categories based on sexual risk behaviour, testing, and HIV prevention questions: (i) any condomless anal intercourse with casual partners in the six months prior to survey; (ii) timing of last HIV test (‘frequent tester’ – less than or equal to a year ago, versus ‘infrequent tester’ – more than a year ago); (iii) at high risk of HIV”. High risk of HIV infection was defined as non-HIV-positive participants who reported condomless anal intercourse with casual partners and no PrEP use in the last six months.

### Statistical Analyses

First, we used descriptive statistics to summarise the demographic and behavioural characteristics of the study population overall and by calendar year of the survey. Then we calculated the uptake of HIV testing at home; the denominator was the total number of non-HIV-positive GBM who completed the survey in the calendar year and the numerator was the number of non-HIV-positive GBM who reported that their last HIV test was at home. There was a potential for GBM to participate in surveys on more than one occasion which violates the assumption of independent observations. We used Poisson regression models with robust estimates of standard errors to assess overall trends in the uptake of home testing overall between 2018 and 2020, and the trends within subgroups, expressed as an incidence rate ratio. HIV test frequency in the last year was adjusted in Poisson regression models.

Additionally, multivariable logistic regression was used to assess correlates of testing at home (versus at a facility) for the participant’s last HIV test. Bootstrapping with 500 repetitions was run to provide unbiased confidence intervals to avoid potential overfitting problems owing to small numbers. The study sample excluded men who had never tested, and only the most recent survey in each jurisdiction (2019 or 2020) was included to ensure the study sample was national. Variables with p-values of < 0.1 in the bivariate logistic regression were included in the multivariable logistic regression. Because three monthly HIV testing is recommended for GBM who use PrEP and they report much higher levels of testing than other GBM [3], we conducted a sensitivity analysis to assess the correlates excluding PrEP users. All analyses were performed using Stata version 14.0 (Stata Corp 2015, College Station, TX; Stata Press).

## Results

### Participant Characteristics

Overall, 24,214 non-HIV-positive men completed GCPS surveys between 2018 and 2020. The median age of participants was 34 years (IQR = 27–44). More than half of the participants were employed full-time (65.0%) and university-educated (57.0%). A third of men were born overseas (30.2%). The majority of participants (61.9%) lived in a suburb with < 5% gay residents. Among those who had casual partners, over half had any condomless anal intercourse with casual partners in the last 6 months (58.3%). Nearly a third (30.6%) were infrequent testers.

### Trends in Participant Characteristics

Between 2018 and 2020, the demographic characteristics of participants remained relatively stable. At the same time, more substantial changes occurred in HIV testing, sexual behaviour and prevention, with increasing trends in the proportion that were infrequent testers (29.4-33.7%, Rate Ratio (RR) = 1.09, 95%CI = 1.06–1.12, *p*-trend < 0.001), reporting any condomless anal intercourse with casual partners (54.2-59.8%, RR = 1.07, 95%CI = 1.04–1.10, *p*-trend < 0.001), and use of PrEP in the last 6 months (20.5-31.2%, RR = 1.19, 95%CI = 1.15–1.23, *p*-trend < 0.001). There was decline in the proportion of men classified as being at higher risk of HIV, reporting any condomless anal intercourse with casual partners and not being on PrEP in the last 6 months(18.9%-13.1%, RR = 0.85, 95%CI = 0.81–0.88, *p*-trend < 0.001) (Table 1 & Supplementary Table 2).

**Table 1:** Socio-demographic and behavioural practices of non-HIV-positive GBM during 2018–2020

Variables	2018	2019	2020	Total
	n = 7846 (32.4%)	n = 8508 (35.1%)	n = 7860 (32.5%)	n = 24,214
Age (median, IQR)	33 (26–44)	33 (27–44)	34 (27–45)	34 (27–44)
Full-time employed	5080(64.8%)	5629 (66.2%)	5031 (64.0%)	15,740 (65.0%)
University education or higher	4336(55.3%)	4909 (57.7%)	4553 (57.9%)	13,798 (57.0%)
Born overseas	2341(29.8%)	2597 (30.5%)	2367 (30.1%)	7316 (30.2%)
Arrived in Australia *≤* 5 years ago (recent migrant) ^†^	NA	925 (10.9%)	843 (10.7%)	1768 (35.6%) ^†^
Living in suburb with < 5% gay residents	4945 (63.0%)	5105 (60.0%)	4754 (60.5%)	14,804 (61.9%)
PrEP use in last 6 months	1609(20.5%)	2347 (27.6%)	2449 (31.2%)	6405 (26.5%)
Had any condomless anal intercourse with casual partners in last 6 months^‡^	2610(54.2%)	3151 (60.8%)	2767 (59.8%)	8528 (58.3%)
At higher risk of HIV infection in last 6 months^§^	1485(18.9%)	1388 (16.3%)	1030 (13.1%)	3903 (16.1%)
Ever tested for HIV	6979(89.0%)	7717 (90.7%)	6921 (88.1%)	21,617 (89.3%)
Infrequent HIV tester^¶^	2309(29.4%)	2444 (28.7%)	2652 (33.7%)	7405 (30.6%)

GBM = gay and bisexual men; IQR = interquartile range;

† Variable only available since 2019

‡ The dominator for CLAIC was restricted to the men who reported had casual partner(s). For the other variables, missing data were included in denominator

§ Higher risk of HIV infection = involved in CLAIC but not using PrEP

¶ Infrequent tester = tested for HIV more than one year ago or never tested

### Trends in Home Testing

The uptake in home HIV testing among non-HIV positive GBM in Australia was low but increased slightly from 2018 to 2020 (from 0.3 to 0.8%, Adjusted Rate Ratio (aRR = 1.54, 95%CI = 1.23–1.92, p-trend < 0.001). The same increasing trend was observed in the sensitivity analysis, when restricted to non-HIV-positive GBM who reported having an HIV test in the last 12 months (Supplementary Table 3). Uptake of home testing increased among subgroups of interest: from 0.4 to 1.2% among migrants (aRR = 1.70, 95%CI = 1.23–2.35, *p*-trend = 0.001); from 0.6% to 1.54% among men living in the suburb areas with a lower concentration of GBM (aRR = 1.42, 95%CI = 1.09–1.85, *p*-trend = 0.01), from 0.4% to 2018 to 1.6% in 2020 among men at higher risk of HIV (aRR = 1.98, 95%CI = 1.27–3.07, *p*-trend = 0.002); and from 0.3 to 0.8% among infrequent testers (RR = 1.75, 95%CI = 1.15–2.66, *p*-trend = 0.009). For recent migrants (data available since 2019), uptake of home testing increased from 1.1% to 2019 to 2.7% in 2020 (RR = 2.58, 95%CI = 1.23–5.38, *p*-trend = 0.012) (Table 2).

**Table 2:** Trends in home HIV testing among subgroups of Australian non-HIV-positive GBM, 2018–2020

Variables	2018 n = 7846 (%)	2019 n = 8508 (%)	2020 n = 7860 (%)	aRR (95%CI)	p-trend
All participants who reported testing at home	26/7846 (0.3%)	43/8508 (0.5%)	59/7806 (0.8%)	1.54 (1.23–1.92)	<0.001
At higher risk of HIV infection in last 6 months^†^	6/1485 (0.4%)	12/1388 (0.9%)	16/1030 (1.6%)	1.98 (1.27–3.07)	0.002
Born overseas	9/2341 (0.4%)	22/2597 (0.9%)	28/2367 (1.2%)	1.70 (1.23–2.35)	0.001
Arrived in Australia *≤* 5 years ago (recent migrant)	NA	10/925 (1.1%)	22/843 (2.7%)	2.58 (1.23–5.38)	0.012
Living in a suburb with < 5% gay residents	19/4945 (0.4%)	31/5105 (0.6%)	36/4754 (0.8%)	1.42 (1.09–1.85)	0.01
Infrequent HIV testers^¶^	7/2309 (0.3%)	11/2444 (0.5%)	22/2652 (0.8%)	1.75 (1.15–2.66)	0.009

†. At higher risk of HIV infection included those who had condomless sex with casual partners in the last six months but were not using PrEP

¶ Infrequent tester = tested for HIV more than one year ago or never tested

*aRR = adjusted risk ratio, HIV test frequency in the last year was adjusted in the model

### Correlates of home Testing

In the multivariate analysis, non-HIV positive men who recently tested at home were more likely to be recent migrants (aOR = 4.71, 95%CI = 2.59–8.56), at higher HIV risk (aOR = 2.17, 95%CI = 1.14–4.12), infrequent HIV testers (aOR = 2.09, 95%CI = 1.15–3.81) (Table 3). In the sensitivity analysis with excluded PrEP users (6 home testers and 2688 facility testers), only being younger (aOR = 0.97, 95%CI = 0.95–0.99) and recent migrants (aOR = 4.76, 95%CI = 2.63–8.64) were associated with the participant’s last HIV test being at home (Supplementary Table 3).

**Table 3:** Socio-demographic and behavioural correlates of non-HIV-positive GBM whose last HIV test was at home versus a facility (2019–2020)

Variables	Tested at home n(%)	Tested in facility-based settings	Crude Odds Ratio (95% CI) (univariate analysis)	Adjusted Odds Ratio (95% CI)
Overall	62	7724		
Age (median, IQR^†^)	29 (25–39)	35 (28–45)	0.97 (0.94–0.99)**	0.98 (0.95–1.01)
Employed full-time				
*No*	22 (35.5%)	2507 (32.5%)	Ref	
*Yes*	40 (64.5%)	5208 (67.4%)	0.88 (0.52–1.48)	
University-level education				
*No*	18 (29.0%)	3029 (39.2%)	Ref	
*Yes*	44 (71.0%)	4688 (60.4%)	1.59 (0.91–2.75)	
Born overseas				
*No*	31 (50.0%)	5362 (69.4%)	Ref	Ref
*Arrived in Australia > 5 years ago*	8 (12.9%)	1570 (20.3%)	0.88 (0.40–1.92)	0.87 (0.38–2.06)
*Arrived in Australia ≤ 5 years ago (recent migrants)*	22 (35.5%)	759 (9.8%)	5.01 (2.89–8.70)***	4.71 (2.59–8.56)***
Living in a suburb				
*<5% gay residents*	39 (62.9%)	4767 (61.7%)	Ref	
*≥5% gay residents*	21 (33.9%)	2868 (37.1%)	0.89 (0.53–1.52)	
At higher risk of HIV infection in last 6 months^‡^				
*No*	46 (74.2%)	6705(86.8%)	Ref	Ref
*Yes*	16 (25.8%)	1019(13.2%)	2.29(1.29–4.06)***	2.17 (1.14–4.12)**
Time since last HIV test				
*Frequent tester*	37 (59.7%)	5695 (73.7%)	Ref	Ref
*Infrequent tester*^¶^	25 (40.3%)	2015 (26.1%)	1.91(1.15–3.18)***	2.09 (1.15–3.81)**

Significant level *<0.1 **<0.05 ***<0.01

† IQR = interquartile range;

‡ At higher risk of HIV infection included those who had condomless sex with casual partners in the last six months but were not using PrEP; Missing data were included in denominator

¶ Infrequent tester = tested for HIV more than one year ago or never tested

## Discussion

This is the first study which has examined changes in home HIV testing among GBM as HIV self-tests became commercially available in Australia. Despite two in three GBM previously reporting that they would like to use HIV self-testing if it was available [21], our study using national behavioural surveillance data shows only a very small proportion of men (fewer than 1 in 100) had recently tested for HIV at home between 2018 and 2020. Uptake of home HIV testing increased slightly after the first HIV self-test kit was made commercially available in Australia (from 0.3% to 2018 to 0.8% in 2020). In addition, our findings suggest that the uptake of home HIV testing was slightly more likely among subgroups of GBM who were most likely to benefit from it.

We found that subgroups of men previously identified as having suboptimal HIV testing uptake or frequency were 2–4 times more likely to report testing at home, including infrequent testers, recent migrants, and men who were at higher risk of HIV. These findings were consistent with previous findings from the FORTH trial that infrequent GBM testers were four times more likely to test for HIV if given free access to HIV self-tests, compared to a control group in which men only had access to clinic-based testing [9]. In addition, follow-up analysis of the same trial showed that sustained use of HIV self-testing was higher among migrants than other participants [22]. The finding that migrants are more likely to use home testing for HIV is encouraging, given that several barriers have been reported that prevent migrants from accessing HIV testing in Australia. These barriers include unfamiliarity with the local health system, distrust of doctors, and concerns about confidentiality, which might potentially explain why a high proportion of late HIV diagnosis are observed among migrants [23]. Although HIV self-tests are generally less sensitive than lab-based tests, they are useful for populations who may have delayed testing or for people who may have been infected months or years ago. Tailored HIV self-testing programs for migrant GBM may therefore improve early HIV diagnosis in this group in Australia [24]. Furthermore, the higher use of home HIV testing among higher risk men is a novel finding and may be related to less frequent clinic attendance compared to PrEP users. In combination, infrequent testers, recent migrants and men who were at higher risk of HIV, who are all priority groups in Australia’s HIV epidemic, comprised 44.1% of all GBM participants in 2020. This emphasizes the potential significance of HIV self-tests in increasing testing uptake among priority groups and helping to control the HIV epidemic in Australia.

The low uptake of self-tests post commercialization, compared to the encouraging findings observed in the FORTH trial, raises questions about the current awareness and accessibility of HIV self-testing among GBM in Australia. A recent discrete choice experiment in Australia highlighted that low cost and having HIV self-test kits available in multiple locations, including pharmacies, was important to GBM [19]. However between December 2018 and October 2021, HIV self-tests in Australia could only legally be purchased online (AU$25 plus shipping costs), one kit at time, and an instructional video was required to be watched before purchasing online, with strict advertising regulations. Encouragingly, informed by the increasing evidence on HIV self-test usability among GBM, the regulations on the distribution of HIV self-test kits were relaxed in Australia and unlimited self-test kits can now be sold in pharmacies and online[25], as in many other counties. Most facility-based HIV testing is free in Australia i.e. there is no charge for the testing itself. However, attending a general practice to see a doctor for testing may attract a fee, if the doctor does not directly bill Medicare for seeing patients, and this may be unaffordable for people on low incomes. The cost of an HIV self-test ($25–30) from a pharmacy may deter those on lower incomes from purchasing a test. HIV community organisations have developed small projects in Australia to offer HIV self-tests for free, with advice provided by peers [26]. Evaluation and expansion of such models to support migrants who face barriers to facility-based testing, and those on lower incomes, would be justified.

The strengths of our study include use of a large repeated national behavioural survey, which collects an extensive range of data on risk behaviours, testing and prevention. There are a few limitations to consider when interpreting our findings. First, the questionnaire asked about the location where the participant’s last HIV test occurred (not every location where testing had occurred in the past year), so it may underestimate the level of home testing. In addition, the questionnaire asked about HIV testing ‘at home’, rather than the specific use of a HIV self-test. For participants in New South Wales, ‘testing at home’ might have involved HIV self-testing or collecting a sample at home to send to a lab for testing [15]. However, as home sampling was not available in the other jurisdictions, our findings are likely to reflect the use of HIV self-tests among GBM in Australia. Furthermore, our analysis is limited by the use of repeated, cross-sectional surveys, which might have potential sampling biases as the characteristics of respondents may change over time. However, to minimize this bias, we consistently recruited large samples using the same methodology across survey rounds. In addition, the Gay Community Periodic Surveys target GBM at higher risk of HIV through gay venues and events, clinics, and online, and are unlikely to be representative of all men who have sex with men in Australia. A more representative sample would feature a broader age range and more participants from regional areas.

## Conclusions

Australia aims to virtually eliminate HIV transmission by 2025. Our study shows that HIV self-testing is not yet contributing substantially to these goals. To increase access, particularly among priority subgroups, HIV self-testing should be made available in more locations, be advertised more widely, and provided at a price point that is affordable. As HIV self-test kits are likely to become available in more pharmacies across Australia, further studies are needed to demonstrate their uptake and acceptability.

GCPS = Gay Community Periodic Surveys; GBM = gay and bisexual men.

### Electronic Supplementary Material

Below is the link to the electronic supplementary material.

Supplementary Material 1

## Data Availability

The datasets generated during and/or analysed during the current study are available from the corresponding author on reasonable request.

## References

[1] Australian Government Department of Health. Eighth National HIV Strategy 2018–2022. 2018.

[2] Joint United Nations Programme on HIV/AIDS. The aids epidemic can be ended by 2030-with your help Geneva. Switzeland UNAIDS; 2016.

[3] Bavinton BR, Grulich AE, Broady T, Keen P, Mao L, Patel P (2020). Increases in HIV Testing frequency in australian gay and bisexual men are concentrated among PrEP users: an analysis of australian behavioural Surveillance Data, 2013–2018. AIDS Behav.

[4] The Australasian Society of HIV VHaSHMA. PrEP Guidelines Update. Prevent HIV by Prescribing PrEP. Sydney; 2019.

[5] Patel PG, Keen P, McManus H, Duck T, Callander D, Selvey C (2021). Increased targeted HIV testing and reduced undiagnosed HIV infections among gay and bisexual men. HIV Med.

[6] Aung ECC, McGregor S, Holt M, Grulich AE, Bavinton BR. Identifying gaps in achieving the elimination of HIV transmission among gay, bisexual, and other men who have sex with men in Australia: the gaps Project Report. Sydney Kirby Institute, UNSW Sydney; 2020.

[7] Gray RT, Wilson DP, Guy RJ, Stoove M, Hellard ME, Prestage GP (2018). Undiagnosed HIV infections among gay and bisexual men increasingly contribute to new infections in Australia. J Int AIDS Soc.

[8] World Health Organization (2016). Guidelines on HIV Self-Testing and Partner Notification: supplement to Consolidated Guidelines on HIV Testing Service.

[9] Jamil MS, Prestage G, Fairley CK, Grulich AE, Smith KS, Chen M (2017). Effect of availability of HIV self-testing on HIV testing frequency in gay and bisexual men at high risk of infection (FORTH): a waiting-list randomised controlled trial. Lancet HIV.

[10] Katz DA, Golden MR, Hughes JP, Farquhar C, Stekler JD (2018). HIV Self-Testing increases HIV Testing frequency in high-risk men who have sex with men: a Randomized Controlled Trial. J Acquir Immune Defic Syndr.

[11] Wang Z, Lau JTF, Ip M, Ho SPY, Mo PKH, Latkin C (2018). A randomized controlled trial evaluating efficacy of promoting a home-based HIV Self-Testing with Online Counseling on increasing HIV Testing among Men who have sex with men. AIDS Behav.

[12] Zhang C, Koniak-Griffin D, Qian HZ, Goldsamt LA, Wang H, Brecht ML (2020). Impact of providing free HIV self-testing kits on frequency of testing among men who have sex with men and their sexual partners in China: a randomized controlled trial. PLoS Med.

[13] Sara Bell JD, Jime Lemoire I, Durkin J, Debattista A, Redmond C, Gilks, Owain Williams. Online HIV self-testing service in Queensland: users, usage and usability, Australasian. HIV/AIDS Conference; Sydney2018.

[14] Williams OD, Dean JA, Harting K, Bath K, Gilks CF (2017). Implications of the on-line market for regulation and uptake of HIV self-testing in Australia. AIDS Care.

[15] Central and Eastern Sydney Primary Health Network. Launching the Dried Blood Spot (DBS) HIV Testing Project [Available from: https://www.cesphn.org.au/news/latest-updates/57-enews/2430-launching-the-dried-blood-spot-dbs-hiv-testing-project.

[16] Regulator-approved. HIV self-testing kits have finally gone on sale in Australia [press release]. ABC.net2019.

[17] Holt M, Lea T, Mao L, Zablotska I, Lee E, de Wit JBF (2017). Adapting behavioural surveillance to antiretroviral-based HIV prevention: reviewing and anticipating trends in the australian Gay Community periodic surveys. Sex Health.

[18] The Kirby Institute. HIV, viral hepatitis and sexually transmissible infections in Australia - Annual Surveillance Report. UNSW Sydney; 2018.

[19] Ong JJ, De Abreu Lourenco R, Street D, Smith K, Jamil MS, Terris-Prestholt F (2020). The Preferred Qualities of Human Immunodeficiency Virus Testing and Self-Testing among Men who have sex with men: a Discrete Choice Experiment. Value Health.

[20] Aung E, Chan C, McGregor S, Holt M, Grulich AE (2020). Identifying gaps in achieving the elimination of HIV transmission among gay, bisexual, and other men who have sex with men in Australia: the gaps Project Report.

[21] Prestage G, Zablotska I, Bavinton B, Grulich A, Keen P, Murphy D (2016). Previous and future use of HIV self-testing: a survey of australian gay and bisexual men. Sex Health.

[22] Zhang YJM, Smith KS, Applegate TL, Prestage G, Holt M, Keen P, Bavinton B, Chen M, Conway D, Wand H, McNulty AM, Russell D, Vaughan M, Batrouney C, Wiseman V, Fairley CK, Grulich AE, Law M, Kaldor JM. Guy RJ The longer-term effects of access to HIV self-tests on HIV testing frequency in high-risk gay and bisexual men: follow-up data from a randomised controlled trial. The Lancet Regional Health-Western Pacific 2021;14. 10.1016/j.lanwpc.2021.100214 PMC8484892 34671752

[23] Marukutira T, Gunaratnam P, Douglass C, Jamil MS, McGregor S, Guy R (2020). Trends in late and advanced HIV diagnoses among migrants in Australia; implications for progress on fast-track targets: a retrospective observational study. Med (Baltim).

[24] Aung E, Blondell SJ, Durham J (2017). Interventions for increasing HIV Testing Uptake in Migrants: a systematic review of evidence. AIDS Behav.

[25] McLay N, Australian Government. Therapeutic goods (Prohibited and Restricted Representations - HIV Self-test) Permission 2021: Department of Health, ; 2021 [updated 14 October 2021. Available from: https://www.tga.gov.au/advert-exempt/therapeutic-goods-prohibited-and-restricted-representations-hiv-self-tests-permission-2021#s5.

[26] ACON, [TEST] YOU, HELPING YOU TEST FOR HIV. 2020 [cited 2021 09/10]. Available from: https://endinghiv.org.au/test-often/book-a-test-at-youtest/.

