# Supplementary material for: National Surveillance of Home-Based HIV Testing Among Australian Gay and Bisexual Men, 2018–2020: Uptake After Commercial Availability of HIV Self-Tests
Source: AIDS Behav. 2023 Jul 13;27(12):4106–13. doi: 10.1007/s10461-023-04124-x (PMC10598086; doi:10.1007/s10461-023-04124-x)
Supplement: Supplementary file 1 — Supplementary Material 1 [file 10461_2023_4124_MOESM1_ESM.docx]

**Supplementary table 1: Timeframe for data collection in Gay Community Periodic Surveys**

| **State** | **Recruitment year and month** | | |
| --- | --- | --- | --- |
|  | **2018** | **2019** | **2020** |
| New South Wales | February-March | February | February * |
| Victoria | January | January | January* |
| Queensland | September-November | September-November | September-December* |
| Western Australia |  | November* |  |
| Australian Capital Territory |  | November-December* |  |
| South Australia | November-December |  | November-December* |
| Tasmania | November |  | October-November* |

*Included in the analysis of correlates of home testing

**Supplementary table 2 Socio-demographic and behavioural practices of non-HIV-positive GBM during 2018-2020**

| **Variables** | **2018** | **2019** | **2020** | **Trend analysis** | |
| --- | --- | --- | --- | --- | --- |
|  | n=7846  (32.4 %) | n=8508  (35.1%) | n=7860  (32.5%) | RR (95%CI) | p-trend |
| Age (median, IQR) | 33 (26-44) | 33 (27-44) | 34 (27-45) | NA | <0.001 |
| Full-time employed | 5080 (64.8%) | 5629 (66.2%) | 5031 (64.0%) | 1.02 (0.99-1.04) | 0.14 |
| University education or higher | 4336 (55.3%) | 4909 (57.7%) | 4553 (57.9%) | 1.05 (1.03-1.07) | <0.001 |
| Born overseas | 2341 (29.8%) | 2597 (30.5%) | 2367 (30.1%) | 1.04 (1.01-1.07) | 0.01 |
| Arrived in Australia *≤* 5 years ago (recent migrant) ^a^ | NA | 925 (10.9%) | 843 (10.7%) | NA |  |
| Living in suburb with <5% gay residents | 4945 (63.0%) | 5105 (60.0%) | 4754 (60.5%) | 1.10 (1.06-1.14) | <0.001 |
| PrEP use in last 6 months | 1609 (20.5%) | 2347 (27.6%) | 2449 (31.2%) | 1.19 (1.15-1.23) | <0.001 |
| Had any condomless anal intercourse with casual partners in last 6 month^b^ | 2610 (54.2%) | 3151 (60.8%) | 2767 (59.8%) | 1.07 (1.04-1.10) | <0.001 |
| At higher risk of HIV infection in last 6 months ^c^ | 1485 (18.9%) | 1388 (16.3%) | 1030 (13.1%) | 0.85 (0.81-0.88) | <0.001 |
| Ever tested for HIV | 6979 (89.0%) | 7717 (90.7%) | 6921 (88.1%) | 1.00 (0.99-1.03) | 0.30 |
| Infrequent HIV tester^d^ | 2309 (29.4%) | 2444 (28.7%) | 2652 (33.7%) | 1.09 (1.06-1.12) | <0.001 |

GBM = gay and bisexual men; IQR = interquartile range;

1. Variable only available since 2019
2. The dominator for condomless anal intercourse with casual partners was restricted to the men who reported had casual partner(s). For the other variables, missing data were included in denominator
3. At higher risk of HIV infection included those who had condomless sex with casual partners in the last six months but were not using PrEP
4. Infrequent tester are the men who have tested for HIV more than one year ago or never tested

**Supplementary table 3 Socio-demographic and behavioural correlates of non-HIV-positive and non-PrEP-using GBM men whose last HIV test was at home or at a facility (2020)**

| **Variables** | **Tested at home**  **n (%)** | **Tested at a health facility**  **n (%)** | **Crude Odds Ratio (95% CI)**  **(univariate analysis)** | **Adjusted Odds Ratio (95% CI)** |
| --- | --- | --- | --- | --- |
|  | 56 | 5036 |  |  |
| Age (median, IQR) | 29 (25-39) | 35 (28-47) | 0.96 (0.94-0.99)*** | 0.97 (0.95-0.99)* |
| Employment status |  |  |  |  |
| *Part-time Employed, unemployed or other* | 19 (33.9%) | 1784 (35.4%) | Ref |  |
| *Full-time Employed* | 37 (66.1%) | 3246 (64.5%) | 1.07 (0.61-1.87) |  |
| Education level |  |  |  |  |
| *Tertiary education or lower* | 16 (28.6%) | 2127 (42.2%) | Ref |  |
| *University education or higher* | 40 (71.4%) | 2894 (57.5%) | 1.84 (1.03-3.29) ** | 1.59 (0.86-2.92) |
| Born overseas |  |  |  |  |
| *No* | 27 (48.2%) | 3539 (70.3%) | Ref | Ref |
| *Arrived in Australia >5 years* | 6 (10.7%) | 1003 (19.9%) | 0.78 (0.32-1.90) | 0.62 (0.33-1.95) |
| *Arrived in Australia ≤*  *5 years*  *(recent migrant)* | 22 (39.3%) | 475 (9.4%) | 6.07 (3.43-10.95)*** | 4.76 (2.63-8.64)*** |
| Living in a suburb |  |  |  |  |
| *<5% gay residents* | 37 (66.1%) | 3324 (66.0%) | Ref |  |
| *≥5% gay residents* | 17 (30.4%) | 1644 (32.6%) | 0.93 (0.52-1.65) |  |
| Had any condomless anal intercourse with casual partners in last 6 months |  |  |  |  |
| *No* | 40 (71.4%) | 4030 (80.0%) | Ref |  |
| *Yes* | 16 (28.6%) | 1006 (20.0%) | 1.33 (0.67-2.64) |  |
| Time since last HIV test |  |  |  |  |
| *Within 1 year* | 32 (57.1%) | 3052 (60.6%) | Ref |  |
| *More than 1 year or never* | 24 (42.9%) | 1974 (39.2%) | 1.16 (0.68-1.97) |  |
| Diagnosed with a sexually transmissible infection in the last 12 months |  |  |  |  |
| *No* | 48 (86.7%) | 4040 (80.2%) | Ref |  |
| *Yes* | 3 (5.36%) | 573 (11.4%) | 0.44 (0.14-1.42) |  |

significant level *<0.1 **<0.05 ***<0.01.

IQR = interquartile range; Infrequent tester = last HIV test more than 1 year; At higher risk of HIV infection included those who had condomless sex with casual partners in the last six months but were not using PrEP.

**Supplemental Table 4. Trends in home HIV testing among subgroups of Australian non-HIV-positive GBM, 2018-2020**

| **Variables** | **2018**  **n=7846 (%)** | **2019**  **n=8508 (%)** | **2020**  **n=7860 (%)** | **aRR (95%CI)** | **p-trend** |
| --- | --- | --- | --- | --- | --- |
| Participants with a lower risk of HIV infection in the last 6 months | 20 (0.3%) | 31 (0.4%) | 43 (0.6%) | 1.45 (1.11-1.88) | 0.006 |
| Australian born GBM | 17/5505 (0.3%) | 21/5871 (0.4%) | 30/5452 (0.6%) | 1.38 (1.01-1.88) | 0.04 |
| Living in a suburb with *≥*5% gay residents | 7/2854  (0.3%) | 12/3257  (0.4%) | 21/2985  (0.7%) | 1.77 (1.15-2.72) | 0.009 |
| Frequent HIV testers | 19/5281 (0.4%) | 32/5949 (0.5%) | 37/5038 (0.7%) | 1.45 (1.11-1.90) | 0.006 |
